# Advantages of omics approaches for elucidating metabolic changes in diabetic peripheral neuropathy

**DOI:** 10.3389/fendo.2023.1208441

**Published:** 2023-11-28

**Authors:** Hideji Yako, Naoko Niimi, Shizuka Takaku, Kazunori Sango

**Affiliations:** Diabetic Neuropathy Project, Tokyo Metropolitan Institute of Medical Science, Tokyo, Japan

**Keywords:** diabetic peripheral neuropathy, omics approach, glycolysis, TCA cycle, pyruvate

## Abstract

Various animal and cell culture models of diabetes mellitus (DM) have been established and utilized to study diabetic peripheral neuropathy (DPN). The divergence of metabolic abnormalities among these models makes their etiology complicated despite some similarities regarding the pathological and neurological features of DPN. Thus, this study aimed to review the omics approaches toward DPN, especially on the metabolic states in diabetic rats and mice induced by chemicals (streptozotocin and alloxan) as type 1 DM models and by genetic mutations (MKR, db/db and ob/ob) and high-fat diet as type 2 DM models. Omics approaches revealed that the pathways associated with lipid metabolism and inflammation in dorsal root ganglia and sciatic nerves were enriched and controlled in the levels of gene expression among these animal models. Additionally, these pathways were conserved in human DPN, indicating the pivotal pathogeneses of DPN. Omics approaches are beneficial tools to better understand the association of metabolic changes with morphological and functional abnormalities in DPN.

## 1 Introduction

The term “omics” indicates a comprehensive discipline in biology, such as genes (genomics), RNAs (transcriptomics), proteins (proteomics), metabolites (metabolomics), and metabolic fluxes (fluxomics). Omics techniques have been utilized to elucidate the phenotype of cells or tissue during development, differentiation, aging, and disease progression (1, 2). In particular, these techniques have helped discover biomarkers for metabolic diseases [e.g., diabetes mellitus (DM), non-alcoholic fatty acid liver disease, metabolic syndrome, obesity] and develop corresponding pathogenesis-based remedies (3, 4). Type 2 DM is characterized by multiple metabolic disorders, such as hyperglycemia, dyslipidemia, dysinsulinemia, and obesity, resulting from genetic and lifestyle-associated factors. Continuous diabetic condition exposure develops and progresses to chronic complications, such as diabetic peripheral neuropathy (DPN), retinopathy, and kidney disease. The increasing use of omics approaches in basic and clinical research has resulted in an improved understanding of the pathobiochemical features of diabetes-related abnormalities (5, 6).

**Figure 1:**
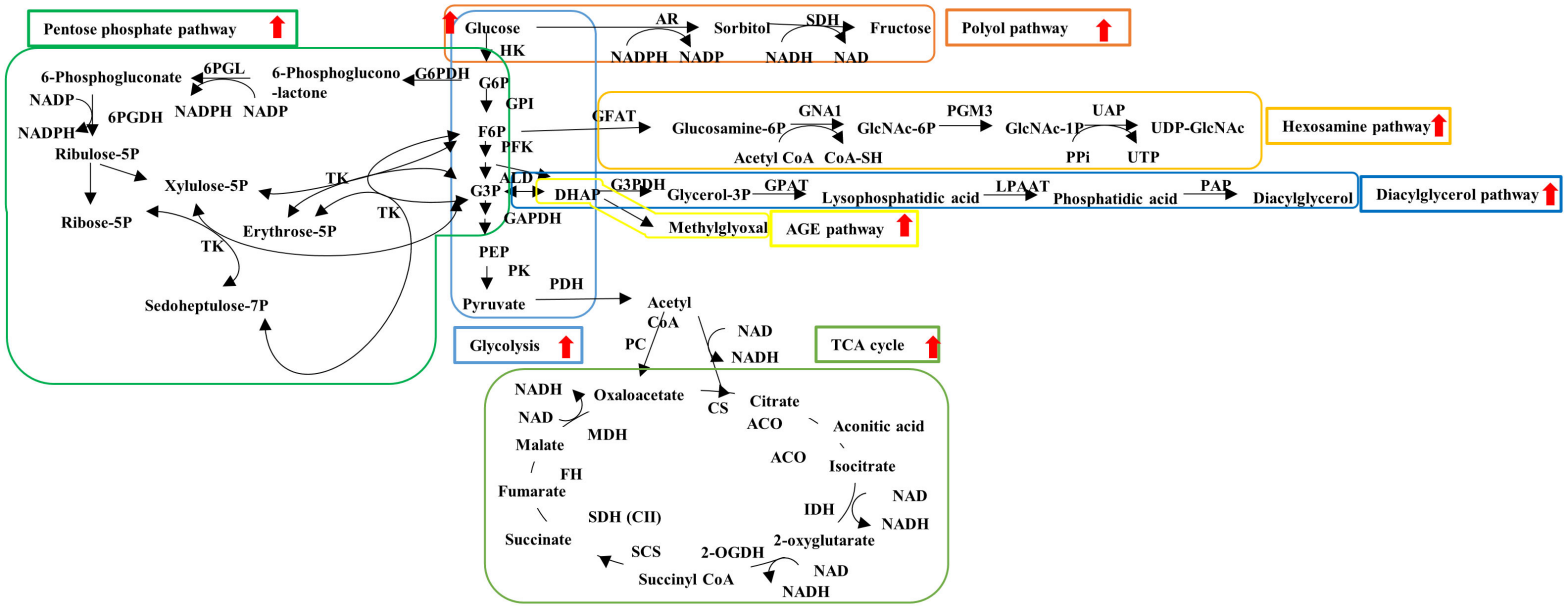
Schematic representation of glucose metabolism. Hyperglycemia enhances the flux into glycolysis (light blue), TCA cycle (light green) and glycolytic collateral pathways such as polyol (brown), hexosamine (orange), diacylglycerol (dark blue), AGE (yellow) and pentose phosphate (dark green) pathways. ACO, aconitase; ALD, aldorase; AR, aldose reductase; CoA, coenzyme A; CS, citrate synthase; FH, fumarate hydratase; F6P, fluctose-6-phosphate; GAPDH, glucose-3-phosphate dehydrogenase; GFAT, glutamine fructose-6-phosphate amidotransferase; GNA1, glucosamine 6-phosphate N-acetyltransferase; GPAT, glycerol-3-phosphate acyltransferase; GPI, glucose-6-phosphate isomerase; G3P, glycerol-3-phaoshate; G3PDH, glycerol-3-phosphate dehydrogenase; G6P, glucose-6-phosphate; G6PDH, glucose-6-phosphate dehydrogenase; HK, hexokinase; IDH, isocitrate dehydrogenase; LPAAT, lysophosphatidic acid acyltransferase; MDH, malate dehydrogenase; 2-OGDH, 2-oxglutarate dehydrogenase; PAP, phosphatidate phosphatase; PC, pyruvate carboxylase; PDH, pyruvate dehydrogenase; PEP, phosphoenol pyruvate; PFK, phosphofructo kinase; PGM3, phosphoacetylglucosamine mutase; PK, pyruvate kinase; 6PGDH, 6-phosphogluconate dehydrogenase; 6PGL, 6-phosphogluconolactonase; RPE, ribulose-phosphate-3-epimerase; RPI, ribose-5-phosphate isomerase; SCS, succinyl CoA synthetase; SDH, sorbitol dehydrogenase; SDH (C II), succinate dehydrogenase (complex II); UAP, UDP-N-Acetylglucosamine pyrophosphorylase. Red allows: Enhancement of flux under diabetes.

DPN is the earliest and most frequent type 2 DM complication and is characterized by distal symmetrical neurological manifestations with axonal length-dependent damage and myelin injury leading to the loss of sensory, motor, and autonomic functions (7, 8). DPN progression increases the risk of infection, foot ulcer, and fatal arrhythmia; however, its pathogenesis remains largely unclear, and therapies better than glycemic control are unavailable to prevent and reduce these unfavorable events (9). Therefore, developing effective pathogenesis-based remedies for DPN has been long awaited. Type 2 DM-related abnormalities, such as hyperglycemia, hyperlipidemia, insufficient vascular supply, and impaired insulin signaling, have been proposed as contributing factors to DPN. In particular, continuous hyperglycemia enhances the fluxes into glycolytic collateral pathways [e.g., polyol pathway, hexosamine pathway, diacylglycerol pathway, advanced glycation end products (AGEs) pathway] in neurons, Schwann cells, and vascular endothelial cells (**Figure 1**).

**Figure 2:**
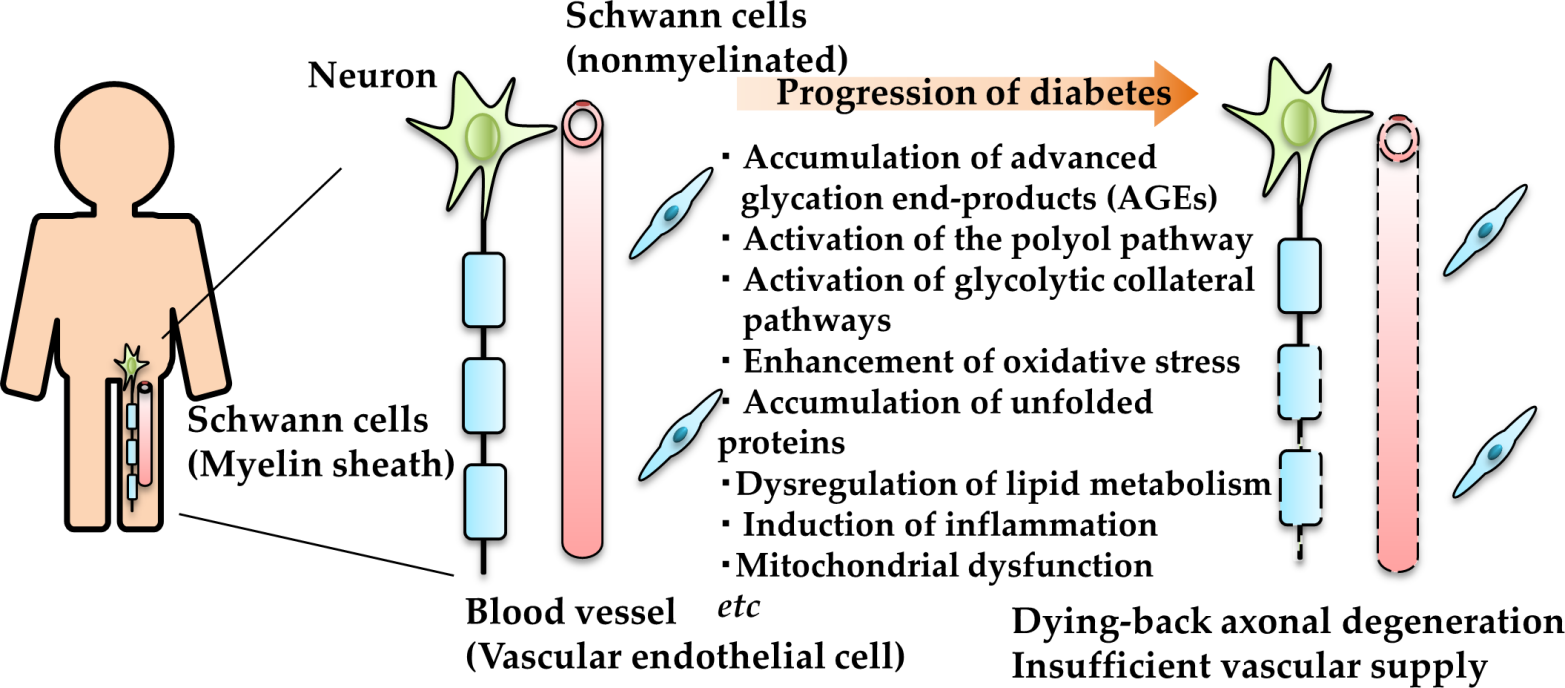
Schematic representation of the pathogenesis of DPN. AGE and unfolded protein accumulation, polyol and other glycolytic collateral pathway activation, and oxidative and endoplasmic reticulum stress enhancement in neurons (green), Schwann cells (blue) and vascular endothelial cells (red) under diabetic conditions leading to dying-back axonal degeneration.

The augmentation of these pathways results in mitochondrial dysfunction, increased reactive oxygen species, AGE accumulation, endoplasmic reticulum stress upregulation, dysregulation of lipid metabolism, induction of inflammatory response, mitochondrial damages, reduced neurotrophic factor synthesis and/or availability etc. (10–14) (**Figure 2**). In particular, the polyol pathway and aldose reductase, a rate-limiting enzyme in this pathway, have been examined in detail. Epalrestat, an aldose reductase inhibitor, has been developed and used for the treatment of DPN in Japan. However, its clinical efficacy was found to be insufficient for the patients with progressive DPN and high HbA1c (15). Because of the limited validity of the single drug (9), the combination of several pathogenesis-based agents toward DPN has been attempted (16, 17).

**Figure 3:**
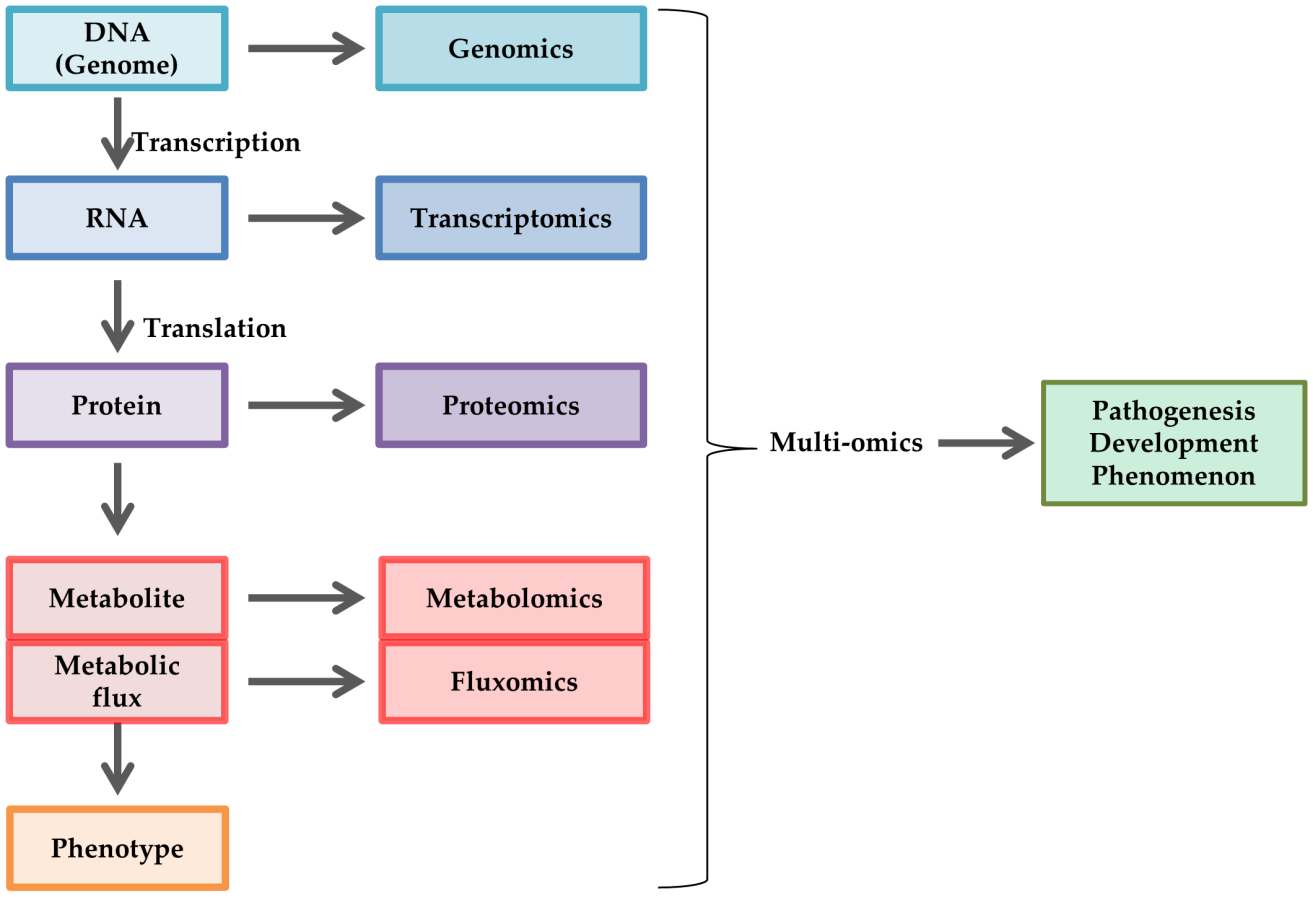
Schematic representation of omics approaches. Omics approaches are utilized to elucidate comprehensive changes in DNA (genomics), RNA (transcriptomics), protein (proteomics), metabolite (metabolomics), and metabolic flux (fluxomics) in cell biology, such as pathogenesis, development, and phenomenon. Multiomics is a combination of these techniques.

Despite these findings, the complicated crosstalk among the pathogenic factors and the different phenotypes induced by hyperglycemia among the cell types make it difficult to understand the pathogenesis of DPN. Omics approaches have been frequently utilized for the study of DPN because of the decided advantages for elucidating the comprehensive biological changes and the relationship among the pathometabolic pathways (**Figure 3**). Because diabetic model animals show different profiles such as strains, procedure of diabetes induction, duration of hyperglycemia etc., it is difficult to fully comprehend the pathometabolism in the peripheral nervous system (PNS). We reviewed the studies regarding metabolic changes in the PNS of the model animals to better understand the pathophysiology of DPN. This review briefly introduces the characteristics of the representative omics approaches, then focuses on the omics studies with cell, animal and human DPN, including our recent study. This review does not address other omics approaches (e.g., glycomics, and microbiomics).

## 2 Omics studies of animal DPN models

Animal models of DM provide insight into the pathogenesis of DPN. Pathophysiological features of DPN in the rodent models of type 1 and type 2 DM have been well-documented (18–20); however, the divergence of metabolic abnormalities among these models makes its etiology complicated. Elucidating the comprehensive changes in the levels of genes, proteins, metabolites, and metabolic fluxes in dorsal root ganglia (DRG) and sciatic nerves (SN) is beneficial to better understand DPN pathophysiology. Here we discuss these changes in diabetic rats and mice induced by chemicals [streptozotocin (STZ) and alloxan (ALX)], genetic mutations (MKR and db/db), and high-fat diet (HFD) (**Supplementary Table 1**). There are abundant reviews of pathophysiological features in these DPN models (18–20). We concisely describe metabolic and neurological lesions in these models.

STZ and ALX are commonly used chemicals to induce nonobese type 1 DM in rodents through pancreatic β cell death induction by alkylation or necrosis, respectively. The animals treated with these chemicals display hyperglycemia and dysinsulinemia with reduced nerve conduction velocity (NCV) and intraepidermal nerve fiber density (IENFD), accompanied by hypo- or de-myelination (18–20). These animals exhibit allodynia in response to thermal and mechanical stimuli (18–20), whereas a recent report shows hypoalgesia in STZ mice (21).

MKR mice, which possess a mutant dominant-negative insulin-like growth factor-1 receptor (KR-IGF-1R) specifically targeted to skeletal muscle, exhibit features of nonobese type 2 DM, such as hyperglycemia, dysinsulinemia and dyslipidemia (22), and reduced NCV, accompanied by hypoalgesia (23). A model of type 2 DM with the phenotypes of obesity, chronic hyperglycemia, pancreatic β cell atrophy, and dyslipidemia consisted of db/db mice generated by homozygous mutation of the leptin receptor. These mice exhibit reduced NCV without myelin damage (18–20). Leptin-deficient BTBR ob/ob mice are also a model of type 2 DM, and exhibit the phenotypes of obesity, hyperglycemia and dyslipidemia, accompanied by DPN phenotypes such as decreases in MNCV, SNCV and IENFD, and increases in hind paw withdrawal latency to a thermal stimulus (24). In the HFD models, rodents are fed with 45%–60% of fat to render obese type 2 DM, such as hyperglycemia, hyperinsulinemia, and dyslipidemia. These models display reduced NCV (18–20). HFD C57BL/6 mice have shown different DPN phenotypes depending on the source and percentage of fat, and duration of feeding. The mice fed in HFD containing 45 and 60% lard for 34 and 31 weeks (final ages of 37- and 36-week-old) exhibited thermal hypoalgesia and decreased motor and sensory NCV and IENFD (25, 26). The mice fed in chow of 54% vegetable oil for 8 weeks (15-week-old at final age) exerted mechanical, but not thermal allodynia, decreased motor, but not sensory NCV, and no significant effects on IENFD (27). The mice fed with 42% anhydrous milk fat exhibit mechanical and thermal allodynia (28). In addition to the different fat sources and percentages, strain (29) and sex (30) of HFD-mice have impacts on pain and insulin resistance of mice, respectively. These studies indicate that various diabetic conditions have differential effects on the PNS; the impact on metabolism in the PNS appears to be far more complicated than expected. We summarized metabolic changes of omics studies in diabetic model rodents (**Supplementary Table 1**).

### 2.1 Genomics

DNA includes information that plays essential roles in living organisms. Genomics is the study of the whole or partial information of the DNA sequences in several organisms, and clarifies the function of DNA sequences (31). The sequence of the human whole genome contains 2.85 billion nucleotides and consists of 20,000–25,000 protein-coding genes (32). Genomics covers genetic variations, such as single nucleotide polymorphism (SNP), rare variants, and copy number variants, which are involved in disease development and progression (31). Therefore, detecting these variations will help elucidate the etiology of diseases and the development of corresponding effective remedies.

Although the correlations between DPN and the SNPs of transketolase (33) and vascular endothelial cell growth factor (34, 35) have been reported in patients with diabetes, it remains unclear how these SNPs are involved in the pathogenesis of DPN.

### 2.2 Transcriptomics

Transcriptomics focuses on RNA profiles in cells and organ under several conditions and diseases. It is also the quantitative analysis for RNAs, including messenger RNA (mRNA) and non-coding RNA such as micro RNA (miRNA). The expression of these RNAs varies by cell type, organ, and environment, in contrast to the ubiquity of the genome. Microarray and Next-generation RNA-sequence (RNA seq) analyses measure the abundance of transcripts via hybridization to defined complementary probes and the abundance of cDNA from the number of counted transcripts, respectively. Because of an unbiased and more sensitive methodology than microarray, RNA seq is considered as an alternative technique to elucidate the comprehensive changes of gene expression (36). RNA seq has been developed for single cell transcriptomics (37) and spatial transcriptomics (38); the former measures the abundance of cDNA in a single cell, while the latter measures cDNA levels and locates the single cells in the tissue. Microarray or RNA seq are described in **Supplementary Table 1**.

Microarray analysis revealed the significant enrichment of gene ontology (GO) terms, such as glycolysis, tricarboxylic acid (TCA) cycle, and fatty acid oxidation in SN of 24-week-old db/db mice as compared with age-matched control mice (39, 40). Guo et al. (41) evaluated mRNAs correlated with the predicted miRNAs using a combination of mRNA and miRNA microarrays. The targeted mRNAs of DRGs of STZ (65 mg/kg intraperitoneal injection (ip))-diabetic Sprague-Dawley (SD) rats with 8 weeks’ duration were significantly enriched in the metabolic processes of fatty acid and lipids, but not glucose, as compared to those of control rats, according to GO terms and the Kyoto Encyclopedia of Genes and Genomes pathway (KEGG pathway). Consequently, mRNAs regulated by miRNAs control the metabolism of fatty acid and lipids, but not glucose, in DRG from STZ-diabetic rats. These findings suggest the alterations of glucose, fatty acid, and lipid metabolism at the gene levels in DRG and SN of both type 1 and 2 DM models. In addition to the DPN pathogenesis elucidation, transcriptomics has also been used in the therapeutic strategies toward DPN. Pioglitazone, an agonist of peroxisome proliferator-activated receptor γ, ameliorates insulin resistance and differentially controls the cellular metabolism in a tissue-specific manner (42). Microarray and RNA seq revealed that genes involved in energy metabolism were enriched, whereas those involved in inflammation were unchanged in SN in db/db mice treated for 11 weeks with pioglitazone, as compared with non-treated db/db mice (43, 44). Pioglitazone treatment also ameliorated intraepidermal nerve fiber density, but not motor and sensory nerve conduction velocities in the mice. Therefore, metabolic regulation by pioglitazone may be effective for the maintenance of small fibers, whereas inflammation may be more involved in large fiber injuries.

The genes associated with inflammatory responses in SNs of male ob/ob mice were enriched as compared with ob/+ mice at 5 weeks, but not 13 weeks of age. In addition, the enrichment of those genes in SNs of ob/ob mice was similar to that of db/db mice (24). The genes associated with regulation of lipase activity and inflammatory response were enriched in SNs of 26-week-old female ob/ob mice, compared with male ob/ob mice (45). There are differences in pathogenesis of DPN between sexes regardless of genetic background. The genes associated with lipid metabolism were enriched in the conversion into a standard diet in SN of HFD (60% kcal lard) and STZ (75 and 50 mg/kg, ip)-induced diabetic C57/BL mice (46). These transcriptomic studies using microarray and RNA seq demonstrated the enrichment of the genes involved in lipid metabolism. The pathways of lipid metabolism are also reversible; therefore, lipid metabolism is one of the therapeutic candidates of DPN.

Single cell RNA seq is useful for analyzing the characterization of single cells. By using the technique, the mechanical allodynia-associated cluster (MAAC) was observed in DRGs of STZ (60 mg/kg, ip)-injected SD rats with mechanical allodynia. The genes enriched in MAAC recapitulated neurofilament, axon, synapse, and receptor-related biological processes, but not metabolic processes. The gene expression pattern of MAAC was close to that of peptidergic nociceptor neuron cluster; whereas MAAC decreased the genes involved in inflammatory response (47). Further studies with regard to these phenomena may clarify the pathobiology of pain symptoms. SNs in HFD (60% high fat)-induced diabetic C57BL/6J mice for 17 weeks’ duration were divided into 4 Schwann cell subpopulations (non-myelinating, myelinating, immature, and repair) and a distinct macrophage cluster. Immature Schwann cells in HFD-mice predicted a transition to myelinating Schwann cell cluster and then macrophage cluster associated with changing the expression of genes involved in lipid metabolism and inflammatory response (48). These findings also imply that lipid metabolism and inflammatory response are deeply involved in the development and progression of DPN.

Furthermore, these pathogenic factors are conserved in SN and DRG among different models of diabetes, thereby being the targets of pathogenesis-based therapy for DPN.

### 2.3 Proteomics

Proteomics is a large-scale study of amounts of peptide and protein, and allows us to identify the comprehensive changes of protein expression depending on cellular physiological states (49). Discordance between the protein level and mRNA level of the molecule occasionally occurs because the amount of protein is influenced by transcriptional regulation, translational efficacy, and proteostasis such as protein turnover (50). Moreover, the amount of intracellular protein in cells reflects these processes; whereas, extracellular protein in cells must be concerned to be loss of protein due to secretion. Therefore, directly assessing the quality and quantity of proteins is important for analyzing cellular profiles.

The amounts of enzymes involved in glycolysis, TCA cycle, and oxidative phosphorylation were upregulated in SN of STZ-diabetic rats with 12 weeks’ duration compared with that of control rats; whereas no significant differences were observed in the amounts of these enzymes in DRG between the two kinds of rats (51). However, another study revealed that the number of mitochondrial proteins associated with oxidative phosphorylation in DRG was significantly downregulated in STZ-diabetic rats with 22 weeks’ duration compared with control rats (52). These findings provide the pathophysiological evidence that DPN lesions progress from distal to proximal portion, and glucose flux may initially increase and then decrease as the duration of diabetes advances. In contrast to STZ-diabetic rats, the amounts of enzymes in charge of glycolysis, TCA cycle, and lipid biosynthesis were downregulated, and those in charge of lipid catabolism were upregulated in both SN and DRG in 21-week-old db/db mice compared with littermate (53). These findings imply that dyslipidemia, which is the characteristic pathometabolism in db/db mice, may be involved in the altered metabolism in the peripheral nervous tissues. Mitochondrial metabolism is also diminished by continuous hyperglycemia, and further declined in addition to dyslipidemia. Mitochondrial differentially expressed proteins in DRGs of mice fed on HFD for 10 weeks were enriched in mitochondrial organization, mitochondrial calcium ion transmembrane transport and metabolism-related pathways (TCA and Respiratory Electron Transport, and Pyruvate metabolism etc.) (54). Allodynia in these mice was accompanied by mitochondrial morphological changes, small fiber degradation, and calcium over-entering into mitochondria in Nav 1.8-positive DRG neurons in response to mechanical stimuli.

These findings demonstrate that the disturbances of glucose and lipid metabolism in diabetic DRGs and SNs serve as one of the causes of DPN. Mitochondrial homeostasis also plays an important role in the metabolism of the PNS under diabetic conditions. It is no doubt that proteomics is a useful technique to understand the protein dynamics; however, the activity of enzymes is also important to elucidate the alteration of metabolic flux in DPN.

### 2.4 Metabolomics

Metabolomics is an effective measure to evaluate the number of metabolites in cells, organ, and biofluids. Metabolites are defined as small molecules (<1,000 Da) besides lipids that have no animal specificity and are more useful for measuring and analyzing molecules than DNAs, RNAs, and proteins (55). Lipidomics is the large-scale study of pathways and networks, and the technique for identification and quantification of molecular lipid species in cells, tissues, and biofluids. Lipids are classified into fatty acyls, glycerolipids, glycerophospholipids, sphingolipids etc. (56); whereas, endogenous metabolites are included in carbohydrates, amino acids, fatty acids etc. (6). These substances are involved in cellular functions (57). Despite some differences, lipidomics is included in metabolomics in this review. Metabolic profiles are closely linked to the phenotypes, such as cell states, biological phenomena, and disease mechanisms, because metabolites are in the downstream of DNAs, mRNAs, and proteins (58). Metabolic profiles, mRNA/protein expression and extracellular environments influence one another. Thus, metabolomic data are comparable beyond animal species (55).

The amounts of glycolytic intermediates in SN were significantly increased in STZ (150 mg/kg, ip)-diabetic C57/BL mice (about 500 mg/dl in blood glucose) with 12 weeks’ duration compared with control mice (59). Another study revealed no significant differences in the TCA cycle metabolite, lactate, and amino acid levels in SN between STZ (60 mg/kg/days, for 5 consecutive days ip)-diabetic C57/BL mice with 12 weeks’ duration and control mice; however, the progression of diabetes (24 weeks) resulted in significant decreases in the TCA cycle metabolite levels and increases in the lactate and sorbitol levels compared with control mice, accompanied by increases in blood glucose levels (from >400 to >500 mg/dl) and development of mechanical and thermal hypoplasia (21). The progression of diabetes with the increase in blood glucose levels correlates with the changed flux of the glycolytic-TCA cycle. These findings suggest the enhanced glycolysis and sustenance of the TCA cycle in SN of STZ-diabetic mice with 12 weeks’ duration, and the subsequent disruption of mitochondrial function with longer hyperglycemia exposure. In contrast to STZ-diabetic mice, no significant differences were detected in the amounts of glycolysis intermediates in SN between STZ (55 mg/kg, ip)-diabetic SD rats with 12 weeks’ duration and control rats (51). The glucose and pyruvate levels in SN of ALX (150 mg/kg, ip)-diabetic SD rats with 26 weeks’ duration were significantly reduced compared with those of control rats, whereas no significant differences were observed between the two kinds of rats in the amounts of glycolytic intermediates, such as glucose-6-phosphate and fructose-1, 6-bisphosphate (60). TCA cycle intermediates such as citrate and malate were increased in tibial nerves of STZ-injected rats with 36 weeks’ duration (61). The reasons for milder metabolic changes in SN of STZ-rats than STZ-mice remain unclear. The amounts of glycolytic and TCA cycle metabolites in DRG and SN in 20- and 24-week-old db/db mice were significantly decreased compared with age-matched control mice (40, 62). These findings suggest more detrimental influences of diabetes on the metabolism of peripheral nervous tissue in db/db mice than in STZ mice, possibly because of the pathogenic background of type 2 DM, such as obesity and dyslipidemia.

Amino acids are catabolized to the glycolysis and TCA cycle intermediates, such as pyruvate, acetyl CoA, oxaloacetate, 2-OG, fumarate and succinyl CoA. Amino acids derived from pyruvate (alanine and serine), acetyl CoA (isoleucine and leucine), oxaloacetate (aspartic acid), 2-OG (histidine and arginine), succinyl CoA (valine and isoleucine), and fumarate (tyrosine) were elevated in the serum of STZ (60 mg/kg/days, for 5 consecutive days ip)-diabetic C57/BL mice with 12 and 22 weeks’ duration (21). Elevated branched-chain amino acids (valine, leucine and isoleucine) decreased the flux into hexosamine pathway and inhibited the activity of pyruvate dehydrogenase (PDH), which mediates the conversion of pyruvate to acetyl CoA via O-glycosylation in heart (63). PDH activity is also regulated in its phosphorylation via pyruvate dehydrogenase kinase (PDK). PDH phosphorylation and PDK activity increased in DRG of STZ (150 mg/kg, ip)-diabetic C57/BL mice with 3 weeks’ duration, and Pdk2/4-deficient mice rendered diabetes by STZ showed milder MNCV and SNCV deficit than wild-type mice (64). These findings suggest that decreases in the TCA cycle intermediates resulting from the reduced amino acid catabolism or intracellular transport and/or the impaired PDH activity in the PNS lead to the development of DPN.

Changes in lipid components in the PNS may play a pathological role in DPN (65, 66). Palavicini et al. (67) assessed myelin and mitochondrial lipids of SN and DRG in obesity-associated (pre)diabetes (db/db and HFD-fed mice) and nonobese diabetes (STZ-injected and MKR mice). Cerebroside, phosphatidylethanolamine, and cardiolipin decreased in SN of obesity-associated mice at 4 weeks (prediabetes), which further decreased at 8 weeks; however, these lipids were not significantly altered in DRG at any time points. The degree of this lipid loss in SN plateaued with DPN progression. Conversely, nonobese diabetic mice did not display significant changes in the amounts of myelin and mitochondrial lipids in SN. Freeman et al. (51) observed changes in triacylglycerol species and reduction in palmitic, stearic, and eicosanoic fatty acids in SN, but not DRG, in STZ (55 mg/kg, ip)-diabetic SD rats. These findings imply that more detrimental lipid metabolism changes in SN than DRG under diabetic conditions might be associated with dying-back axonal degeneration or distal axonopathy in DPN. Moreover, the divergence of metabolic abnormalities among diabetic animal models may be attributable to pathogenic backgrounds, such as obesity, dysinsulinemia, and dyslipidemia. HFD (60% lard)- and combination with HFD and STZ-treated mice elevated triglycerides in SN, accompanied by the increased expression of genes encoding enzymes required for triglyceride synthesis. These increases and DPN lesions were ameliorated by conversion to a standard diet in both mice (46). These findings suggest that reversion of HFD to standard diet ameliorates DPN pathology. Metabolomic studies reveal that hyperglycemia induced by STZ and ALX tends to increase intermediates of glycolysis and TCA cycle; whereas, hyperglycemia and dyslipidemia induced by HFD and genetic effects (db/db and MKR) tend to decrease intermediates of glycolysis and TCA cycle. These findings imply that dyslipidemia is a crucial factor in the development and progression of DPN.

### 2.5 Fluxomics

Metabolomics is an analytical method that identifies metabolite levels at a specific point in time; however, metabolites are consumed and produced in specific and complicated pathways. Metabolic flux is the net result of gene and protein expression, enzyme activity and metabolite concentration, determining physiological cell phenotypes (68). By using isotope-labeled metabolites, fluxomics provides insight into how metabolites of interest change and link through metabolic pathways. The technique is useful for tracing the substances derived from the targeted metabolites in metabolic pathways.

The metabolomic analysis results represent the metabolite levels under static situations; however, almost all the metabolites are converted into others via specific pathways. Therefore, metabolic flux (fluxomics) evaluation is important to elucidate the pathogenesis of DPN. The amounts of TCA cycle intermediates derived from isotope-labeled glucose in SN of db/db mice were significantly lower than those of control mice, but without significant differences in the amounts of glycolytic intermediates between the two kinds of mice. The amounts of TCA cycle intermediates derived from isotope-labeled pyruvate in SN were significantly increased in diabetic animals compared with control littermates (40). These findings indicate that diabetic SN may increase the uptake of pyruvate and/or its utilization for ATP production rather than glucose. Metabolites originating from isotope-labeled palmitate significantly increased in acylcarnitine, citrate, and malate, but decreased in succinate in SN under diabetes (40). These findings imply that either SN utilizes priority for fatty acid to pyruvate in the TCA cycle, or incompletely conducts β oxidation under diabetes. The fluxomic findings indicate that ATP production in SN of db/db mice may depend on pyruvate.

## 3 Omics studies of human DPN

### 3.1 Transcriptomics/epigenomics

In addition to diabetic model rodents, transcriptome data obtained by human sural nerve specimen were classified into two groups, such as progressive DPN (a loss of myelinated fiber density > 500 fibers/mm^2^) and non-progressive DPN (a loss of myelinated fiber density < 100 fibers/mm^2^) (69). The nerves in progressive DPN were enriched in the up-regulated genes of defense and inflammation responses, whereas those in progressive DPN were enriched in the down-regulated genes of glucose and lipid metabolism and peroxisome proliferator-activated receptor signaling pathway, compared with those in non-progressive DPN. By using microarray data and a bioinformatic approach, McGregor et al. (70) reported that transcriptional pathways in SNs were conserved between human and mice models (STZ, db/db and ob/ob) of diabetes. That study indicates the enrichment of lipid metabolism in these samples. Dysmetabolism and inflammation probably serve as DPN lesions, while both microarray and RNA seq analyses reveal enrichment of these genes in the PNS of patients and mice with diabetes. Transcriptomic studies described above indicate that dysregulation of glucose and lipid metabolism and inflammation in diabetic DRGs and SNs are prominent pathophysiological features in human and rodents, and are conserved among these species.

Genetic modification is defined as changes of gene expression without alteration of DNA sequence, and is classified into DNA methylation and Histon modification. DNA methylation is heritable, state and reversible attachment of methyl group on cytosine residue adjacent to guanine residue (71), whereas Histon modification is post-transcriptional attachment of acetyl group etc. (72). The early intensive glycemic control decreased in the risk of diabetic complications including DPN, indicating the metabolic memory (73) that may result from the continuous hyperglycemia-induced oxidative stress, inflammation, AGEs and epigenomic modification (74).

By using RNA seq, Hall et al. (75) investigated the genes in human DRGs differentially expressed between DPN patients and non-diabetic subjects. Genes involved in inflammatory pathways were significantly upregulated in DRGs of DPN patients, compared with those in non-diabetics. Combination analyses with RNA seq and DNA methylation seq in human sural nerves revealed that genes involved in immune response were enriched depending on HbA1c levels (76). These transcriptomic data from diabetic patients and model animals indicate that lipid metabolism and inflammation may be one of the therapeutic targets based on the pathogenesis in DPN.

In contrast to the intensive studies with single RNA seq, there are no reports in human DRGs or SNs of DPN using spatial RNA seq. Clusters of human (77) and mice (78) DRG neurons are similar to the profile of gene expression. However, the specific distribution of subtypes of DRG neurons was not observed in human lumber DRG (77). These findings indicate that the data of diabetic model animals contributes to elucidating pathogenesis of human DPN.

### 3.2 Metabolomics

Condensation between palmitoyl-CoA and serine by serine-palmitoyltransferase is the first and rate-limiting step in *de novo* sphingolipid synthesis, whereas substitution of alanine for serine in this step produces 1-deocyshingolipids. Plasma serine concentration is reduced in mice (79) and human (80) with type 2 DM, and STZ-treated rats (81). Serine supplementation to HFD-fed mice and STZ rats restored DPN lesions and deoxydihydroceramides in paw skin (79, 81). Serum 1-deocyshingolipids may be a candidate of clinical biomarker/mediator in human DPN, and serine supplementation is a therapeutic target on DPN.

## 4 Omics studies in cultured cells under hyperglycemic conditions

Fluxomic study indicates that pyruvate is important for ATP production in SN of db/db mice (40). The decreased activity of pyruvate kinase, an enzyme catalyzing the conversion of phosphoenolpyruvate to pyruvate in glycolysis, was correlated with the progression of diabetic nephropathy (82). Pyruvate administration into STZ-induced diabetic animals restored hyperglycemia (83), retinopathy (84), and nephropathy (85) by anti-oxidative and anti-inflammatory activity (84, 85). Serum pyruvate concentrations in STZ-induced diabetic rats with cognitive dysfunction were reduced compared with those in age-matched control rats (86). Additionally, patients with DM with chronic complications displayed diminished serum pyruvate levels compared with healthy controls and patients with DM without complications (87). These studies inspired us to investigate the involvement of decreased pyruvate supplementation in DPN pathogenesis.

**Figure 4:**
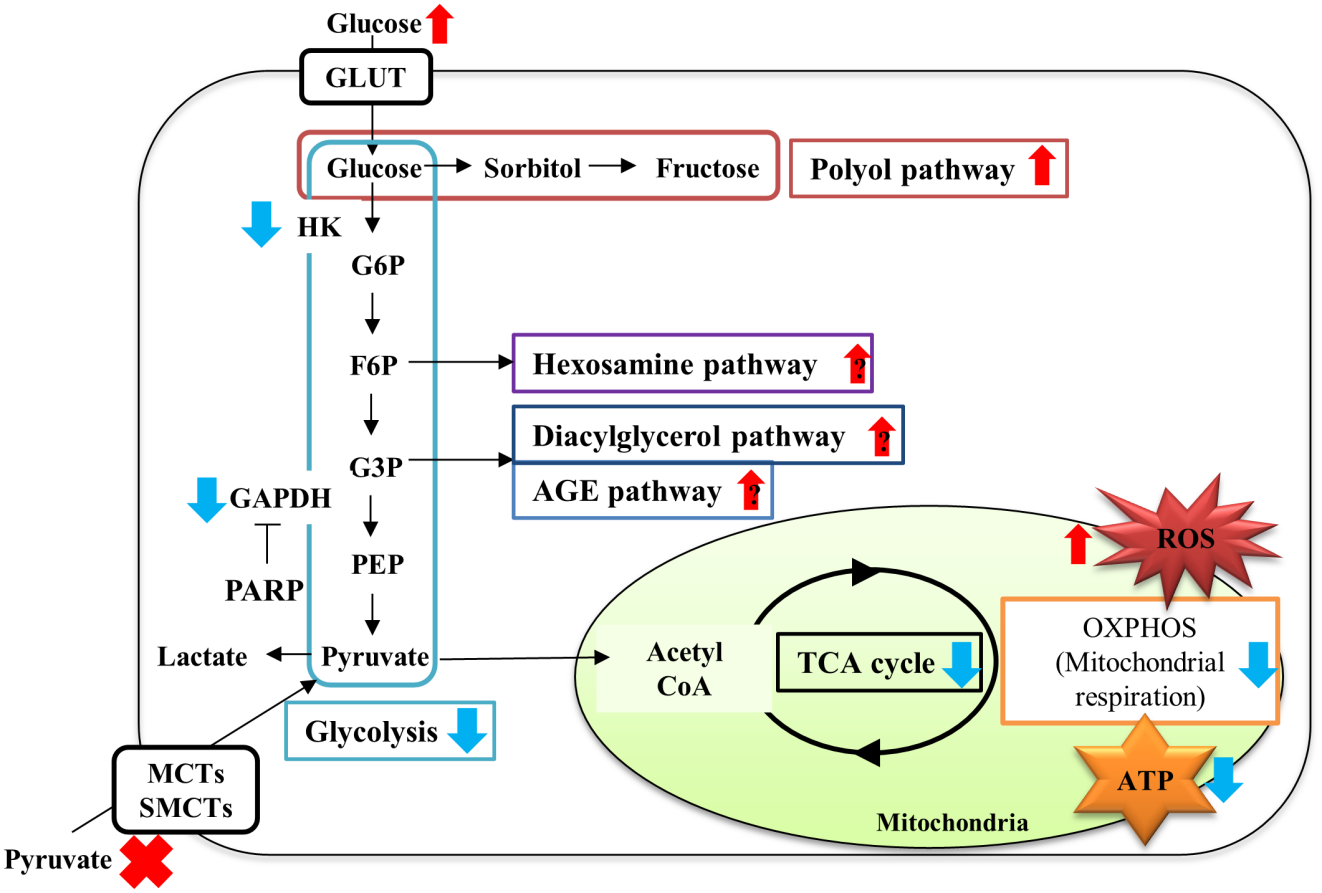
Schematic diagram of metabolic disturbances of IMS32 cells under high-glucose pyruvate-starved conditions. Pyruvate starvation under high-glucose conditions induced decreases in glycolysis (light blue box)-TCA cycle (black circle) flux, and mitochondrial respiration (orange box) and ATP production, and increases in the fluxes of glycolytic collateral pathways (polyol pathway; red, Hexosamine pathway; purple, Diacylglycerol pathway; dark blue, AGE pathway; blue), leading to ATP depletion. G6P: glucose-6-phosphate, F6P: fructose-6-phosphate; G3P: glyceraldehyde-3-phosphate; PEP: phosphoenol pyruvate. Red upper arrows: upregulation; blue downer allows: downregulation.

Our recent study (88) revealed that the absence of exogenous pyruvate under high-glucose (>10 mM) conditions evoked rapid and massive cell death of primary cultured adult rat DRG neurons and immortalized mouse Schwann cells (IMS32). Comprehensive changes in gene expression and metabolites were assessed using a combination of microarray and metabolomic approaches to evaluate the effects of pyruvate starvation on glucose metabolism in IMS32 cells under high-glucose conditions. The absence of pyruvate under high-glucose conditions failed to alter mRNA expression of glycolysis and TCA cycle enzymes; whereas it increased glycolytic intermediates, such as fructose 1,6-bisphosphate and glyceraldehyde-3-phosphate and decreased TCA cycle intermediates, such as citrate, 2-oxoglutarate, and fumarate, compared with the presence of pyruvate. The state of glycolysis and mitochondrial respiration of IMS32 cells under these conditions were evaluated using an Extracellular Flux Analyzer, in addition to these omics approaches (88). High-glucose and pyruvate-starved insults induced swift and irretrievable reductions of mitochondrial respiration and ATP production and a rapid escalation and subsequent steep glycolytic flux reduction. Reduced glycolytic flux under these conditions can be attributable to hexokinase activity suppression via mitochondrial damages (89), and glyceraldehyde 3-phosphate dehydrogenase activity suppression via enhanced poly (ADP-ribose) polymerase activity and its poly (ADP) ribosylation (88). These changes resulted in enhancing flux into glycolytic collateral pathways such as polyol, hexosamine, diacylglycerol and AGE pathways (90–92). These results indicate that exogenous pyruvate maintains glucose metabolism under hyperglycemia via sustaining the activity of glycolytic and TCA cycle enzymes without altering their gene expression (**Figure 4**).

Inflammation induced by proinflammatory macrophage (M1) infiltration is one of the pathogeneses of DPN (93), and is activated via AGEs-RAGE axis in macrophages (94). Macrophage polarization to M1 is regulated by PDK2/4 activation (95). Consistent with these findings, pyruvate plays a crucial role in the maintenance of glucose metabolism (88) and anti-inflammation activity (96) under diabetic conditions. Although further study is needed to validate the mechanisms of diabetes-induced inflammation in macrophages of SN, pyruvate may be one of the therapeutic candidates for DPN based on its pathophysiological features.

## 5 Discussion

Omics approaches are advantageous for elucidating the pathogenesis of DPN, indicating the different phenotypes in diabetic animals, such as STZ-induced, db/db, and HFD-fed rodents. Moreover, protein and metabolic changes in SN are more sensitive to diabetic conditions than those of DRG, corresponding to the cause of DPN, such as distal axonal damage. Multiomics approaches are not always correlated; thus, these discrepancies may be attributed to the regulated hierarchical levels, such as transcriptional regulation and post-transcriptional modification. Metabolic changes under diabetic conditions are controlled in the hierarchical levels of mRNA, protein and metabolism, indicating that amelioration of metabolic alterations in diabetes is necessary to control the level of gene expression.

Accumulated evidence obtained by omics studies will help clarify the different pathogenesis of small and large fibers, resulting from metabolic alteration and elevated inflammation respectively, in SN and DRG in diabetic animal models. Lipid dysmetabolism and inflammation are conserved between human and rodents’ DPN; therefore, these responses may be an important pathogenesis and potent therapeutic target in human DPN. There are accumulating omics studies in DPN. However, there were fewer genomic and epigenomic studies of DPN, and no findings of histone modification. Further studies regarding epigenomics are required to elucidate the pathogenesis of DPN.

## Author contributions

Conceptualization, Writing-original draft preparation: HY. Writing-review and editing: NN, SK, KS. Funding acquisition: HY, SK. All authors contributed to the article and approved the submitted version.

## Supplementary material

Supplementary Table 1 Omics studies regarding the changes of mRNA, protein, metabolite, and metabolic flux in SN and DRG of diabetic animals. ALX, alloxan; db/db, homozygous mutation of the leptin receptor; ob/ob, Leptin-deficient BTBR ob/ob mice; DRG, dorsal root ganglia; HFD, high-fat diet; MKR, transgenic mice expressing a mutant dominant-negative insulin-like growth factor-1 receptor (KR-IGF-1R) specifically targeted to skeletal muscle; SD, Sprague-Dawley; SN, sciatic nerves; STZ, streptozotocin; TCA, tricarboxylic acid; ↑, upregulation; ↓, downregulation; NS, no significant differences. * Enrichment of gene ontology (GO) terms and Kyoto Encyclopedia of Genes and Genomes (KEGG) pathways.
